# Multiple risk behaviour in adolescence and socio-economic status: findings from a UK birth cohort

**DOI:** 10.1093/eurpub/cku078

**Published:** 2014-06-23

**Authors:** Ruth R Kipping, Michèle Smith, Jon Heron, Matthew Hickman, Rona Campbell

**Affiliations:** School of Social and Community Medicine, University of Bristol, BS8 2BN, Bristol, UK

## Abstract

**Background.** Patterns of risk behaviour during teenage years may vary by socio-economic status (SES). We aimed to examine possible associations between individual and multiple risk behaviours and three measures of SES in mid-adolescence. **Methods.** The sample (*n* = 6406) comprised participants from the Avon Longitudinal Study of Parents and Children, a UK birth cohort. Thirteen risk behaviours spanning sexual health, substance use, self-harm, vehicle-related injury, criminality and physical inactivity were assessed in mid-adolescence (age 15–16 years). Associations between three measures of SES (maternal education, household income and parental social class) and (i) individual risk behaviours and (ii) the total number of risk behaviours were examined. **Results.** For a one-category reduction in social class, maternal education or income, the odds of having a greater number of multiple risk behaviours increased by 22, 15 and 12%, respectively. At the individual level, there was evidence of a strong relationship with decreasing SES across all three measures of SES and criminality, car passenger risk, TV viewing, scooter risk, early sexual behaviour and weekly tobacco use but insufficient evidence of a relationship for physical inactivity, cycling without a helmet and illicit substance use. There was weak evidence of association between SES and hazardous drinking, self-harm, cannabis use and unprotected sex, but this was not consistent across the SES measures. **Conclusion.** The association between multiple risk behaviours and SES suggests that prevention strategies should apply the principal of proportionate universalism with a focus on more deprived populations, within a population-wide strategy, to prevent widening of social inequalities.

## Introduction

Risk behaviours in adolescence, such as alcohol intake, substance use, poor diet, physical inactivity and unprotected sex, are common.^1^ The frequency of many of these behaviours increases through adolescence and can continue into adulthood with consequent morbidity and premature mortality.^2^ While many studies examine the clustering of one or two behaviours^3^^,^^4^, few studies have examined a wide range of behaviours.

Many health behaviours in adults differ by socio-economic status (SES), with lower SES associated with increased numbers of unhealthy behaviours.^5^^,^^6^ Yet, in adolescence, such patterning is not always found, with low SES sometimes increasing, decreasing or being unrelated to risk behaviours.^7–10^ Social patterning of behaviour can also change over time, as illustrated by the history of smoking behaviour that started in higher SES groups but the prevalence of which first declined in these groups.^11^ Further, different measures of social class may show different relationships with risk behaviours. For example, the knowledge and skills attained through education may increase health literacy and make individuals more receptive to health education messages, and therefore, maternal education may influence the attitudes of offspring regarding the value of health and engagement in risk behaviours.^12^

A number of competing explanations have been advanced for why risk behaviours in adolescents may be socially patterned. Greater access to support,^9^ having more to lose^9^ and being more concerned about the future^13^ have been suggested as reasons why social position is protective against engagement in risk behaviours. Conversely, it has been proposed that greater access to financial resources may reduce or reverse social patterning for some risk behaviours, e.g. alcohol and substance use.^13^ It has also been hypothesized that risk behaviours are related to general developmental processes, which affect all young people irrespective of SES.^9^ An understanding of the relationship between SES and multiple risk behaviours (MRBs) is important to explore these hypotheses and inform whether interventions to reduce harm should be provided at a population level, should be targeted at population subgroups or should follow the concept of proportionate universalism advocated by Marmot.^14^ Proportionate universalism is suggested as necessary as ‘To reduce the steepness of the social gradient in health, actions must be universal, but with a scale and intensity that is proportionate to the level of disadvantage’ (p. 10).^14^

Using data from a prospective UK birth cohort, this article aims to explore whether there is an association between three measures of SES and engagement in single and multiple risk behaviours in adolescents and, if so, to establish the direction and strength of these associations.

## Methods

### Participants

The participants are from the Avon Longitudinal Study of Parents and Children (ALSPAC), a birth cohort study in the south-west of England. The core sample of ALSPAC includes 14 541 women who were expecting to deliver infants between 1 April 1991 and 31 December 1992 in the former county of Avon, UK^15^: 13 988 infants were alive at 1 year of age, of whom, 13 796 were singletons or first-born twins. More information on the ALSPAC study is available at http://www.alspac.bris.ac.uk. Ethical approval was obtained from the ALSPAC ethics and law committee and the local research ethics committees.

### Risk behaviours

Thirteen indicators of distinct risk behaviours were derived from responses to questions collected either during a clinic visit (median age 15 years 5 months) or a postal questionnaire (median age 16 years 7 months). Of our starting sample of 13 796 singletons/first-born twins, 10 615 participants were invited to the 15 year clinic and/or sent the 16-year questionnaire, and hence, this sample had the potential to provide risk behaviour data for these analyses. Risk behaviours cover the domains of sexual health, criminal and antisocial behaviour, substance use, self-harm, vehicle-related injury risk and physical activity. Further detail on these measures can be found in Supplementary Appendix 1. Binary indicators were used for all behaviours with the choice of threshold for dichotomization influenced by the discrete nature of some behaviours (e.g. sexual risk or cycle-helmet wearing) or by there being a level of engagement likely to infer risk (e.g. alcohol drinking or physical inactivity).

### Socio-economic status

The three measures were derived from questionnaires administered to the main carer. ‘Maternal educational attainment’ was collected during pregnancy and is categorized into below O-level (a national examination at age 16 years); O-level or equivalent; A-level (a national secondary education higher examination taken at age 18 years) or equivalent; University Degree. ‘Household equivalized income’ consists of quintiles of household’s disposable income assessed when the child was aged between 2 and 4 years. Based on declared income, the measure incorporates additional income obtained through housing and council tax benefits and is then adjusted for family size and composition.^16^ ‘Parental social class’ was assigned according to the Registrar General’s Social Class classification based on occupation for the highest social class of either parent. A four-category measure was derived from the original six categories: I (professional), II (managerial/technical), IIINM (skilled non-manual) and IIIM to V (skilled manual, partly skilled and unskilled).

### Statistical methods

Of the 10 615 participants aforementioned, 6470 (61%) either attended the clinic or returned the postal questionnaire. Because of a small amount of non-response ‘within’ these two data-sweeps, a sample of 6406 was available comprising young people who provided information on one or more risk behaviours. Within this sample, each risk behaviour suffered from ∼25% missing data. In all, 41.5% of the sample had complete risk behaviour data, 28.9% had 1–3 missing values, 5.1% had 4–6 missing values, 22.3% had 7–10 missing values and 2.2% had 11 or 12 missing values. We refer to this sample of 6406 as the ‘imputed sample’, and we used a data imputation routine to deal with the partial missingness present in these data (more details can be found below). Imputation was not carried out for those young people with no risk behaviour data recorded at either 15 or 16 years because of the relative sparseness of auxiliary information within this sample. As a comparison with the results based on imputed data, a ‘complete-case analysis’ was also performed, focusing on the subset providing a complete set of risk behaviours and SES measures (*n* = 2346).

#### Binary outcome models

The initial analysis consisted of a series of logistic regression models using each of the 13 risk behaviours as a separate binary outcome. Each risk behaviour was regressed, in turn, on each of the three measures of SES, producing 39 univariable regression models. These analyses were followed by a set of 13 multivariable regression models in which the three measures of SES were mutually adjusted. Of interest were the simplest (most parsimonious) models, which would describe the association between SES and each risk behaviour outcome. Likelihood ratio tests were used to compare models treating SES as categorical with models in which SES was treated as linear. Owing to the somewhat arbitrary metric for the three SES measures, our focus was on deviations from a monotonic association between exposure and outcome (e.g. a J-shaped relationship). For all outcomes and exposures listed, models with a linear relationship were deemed adequate so these models are the focus of the findings presented.

#### Ordinal outcome models

Subsequent analysis examined a composite measure derived from the total number of behaviours. The distribution of total risk behaviours measure for the complete cases sample was highly positively skewed with a median and mode of two behaviours. This lack of normality and a number of low cell counts led to this measure being collapsed into a four-category ordinal variable based on grouping the number of risk behaviours as follows: 0–1, 2–3, 4–6 and 7–13. Once again, linearity of each SES exposure was assessed and a linear relationship was deemed adequate for each SES exposure, as also was the proportional odds assumption for the ordinal outcome.

#### Multiple imputation

Multivariate imputation by chained equations^17^ was carried out using the ‘ice’ routine^18^ in Stata version 11.2 MP2.^19^ This approach is based on the Missing At Random assumption, i.e. that conditional on the data included in the imputation model, there should be no systematic differences between observed and missing values. Additional auxiliary variables were included in the imputation routine such as gender, indicators of family adversity and prior measures of sedentary behaviour, criminal activity and substance use. Monte Carlo errors^17^ were used to compare the results obtained when imputing 25, 100 and 250 data sets. Imputed results shown have been pooled across the 250 datasets. Supplementary Appendix 2 shows a comparison of the estimates obtained with and without imputation.

## Results

Participants were split into three subsamples: (i) the complete case subsample (*n* = 2346), (ii) the cases who provided a ‘partially complete’ set of responses (*n* = 4060) and (iii) those cases with no risk behaviour data (*n* = 7390). There was strong evidence (*P*< 0.001) for an association between level of response and all three SES measures (table 1). The complete case subsample had higher proportions of adolescents from higher SES backgrounds with the partial responders falling midway between the complete responders and non-responders.

**Table 1:** Relationship between SES measures and amount of data provided

SES measure	*n*	Complete cases^a^ (*n* = 2346)	Partial responders^b^ (*n* = 4060)	No risk behaviour data^c^ (*n* ≤ 7390)
Parental social class				
Professional	1513	490 (20.9%)	544 (15.2%)	479 (8.8%)
Managerial and technical	4744	1109 (47.3%)	1568 (43.9%)	2067 (38.0%)
Skilled non-manual	2897	521 (22.2%)	899 (24.9%)	1487 (27.3%)
Skilled manual and lower	2201	226 (9.6%)	570 (16.0%)	1405 (25.8%)
Maternal educational attainment				
Degree	1576	542 (23.1%)	556 (14.5%)	478 (7.9%)
A level	2760	696 (29.7%)	1000 (26.0%)	1064 (17.5%)
O-level	4241	792 (33.8%)	1332 (34.7%)	2117 (34.9%)
<O-level	3682	316 (13.4%)	956 (24.9%)	2410 (39.7%)
Household equivalized income				
Top 20%	1994	667 (28.4%)	712 (21.0%)	615 (15.0%)
Upper middle 20%	1962	590 (25.2%)	677 (20.1%)	695 (16.9%)
Middle 20%	1952	461 (19.7%)	692 (20.6%)	799 (19.5%)
Lower middle 20%	1945	390 (16.6%)	652 (19.4%)	903 (22.0%)
Lowest 20%	1963	238 (10.1%)	633 (18.8%)	1092 (26.6%)

Percentages shown are column percents.

^a^Cases with measurements of all 13 MRB outcomes and all 3 SES measures.

^b^Cases with at least 1 MRB outcome and at least 1 SES measure not missing.

^c^Cases missing all 13 MRB measures (*n*’s vary because of incomplete data on SES for this sample).

Table 2 shows the prevalence of each single risk behaviour for the complete case and imputed sample. Participation in most risk behaviours increased following imputation. The most common single risk behaviour was physical inactivity, followed by criminal and antisocial behaviour and hazardous alcohol drinking. Behaviours such as cannabis use, tobacco smoking, illicit drug and solvent use and unprotected sex were less common, and tended to be undertaken by those individuals engaging in a higher number of total behaviours. Only 4% of adolescents did not engage in any of the risk behaviours.

**Table 2:** Prevalence of single risk behaviours by complete case and imputed sample

Risk behaviour	Complete case sample (*n* = 2346) (in %)	^a^Imputed sample (*n* = 6406) (in %)
Physical inactivity	74.1	74.1
Criminal/antisocial behaviour	42.2	46.2
Hazardous alcohol drinking	34.5	35.6
Car passenger risk	27.8	31.2
Cycle-helmet risk	24.4	25.3
Daily TV viewing (3+ h)	19.7	22.0
Self-harm	19.1	18.6
Scooter risks	16.8	19.7
Sex before age 16 years	13.4	17.3
Tobacco smoking (weekly)	10.0	13.6
Cannabis use	9.4	10.8
Drug/solvent use	4.4	5.5
Unprotected sex	1.4	1.9

^a^Average estimated prevalence of each risk behaviour within the imputation sample. The majority of the risk behaviours increase following the inclusion of partial responders. The exception is self-harm, which is considerably more prevalent in females, whereas incomplete response is more common in males.

### SES and individual risk behaviour

Table 3 shows the linear effect of each SES measure both unadjusted and adjusted for the other two measures. While there is a consistent association across different SES measures for some risk behaviours, e.g. antisocial behaviour and TV viewing, which increase with reducing SES, there are other behaviours for which the pattern is less consistent, for instance, cycle-helmet risk, which ‘increases’ with decreasing household income but ‘decreases’ for decreasing maternal education. A similar pattern is observed for cannabis use, which is more common for those with less income and also those with a higher maternal education. Hazardous alcohol use is the only behaviour ‘negatively’ associated with household income. Finally, it is worth noting that for some behaviours, for example, cycle-helmet use, physical activity and drug/solvent use, there is little evidence of social patterning in this sample. The associations for unprotected sex are also surprising; in recent work,^20^ we have shown that while early sexual behaviour was strongly related to SES, there was little patterning for sexual readiness, of which unprotected sex is one aspect.

**Table 3:** Linear association between decreasing SES and each risk behaviour in mid-adolescence (imputed data, *n* = 6406)

Risk behaviour	Parental social class		Maternal education		Household equivalized income	
	Unadjusted	Mutually adjusted	Unadjusted	Mutually adjusted	Unadjusted	Mutually adjusted
Physical inactivity	1.03 [0.96, 1.10] *P* = 0.49	0.99 [0.90, 1.08] *P* = 0.75	1.07 [1.00, 1.15] *P* = 0.04	1.09 [1.00, 1.18] *P* = 0.04	[0.96, 1.06] *P* = 0.64	0.99 [0.94, 1.05] *P* = 0.74
Criminal/anti-social behaviour	1.10 [1.03, 1.17] *P* = 0.002	1.06 [0.99, 1.15] *P* = 0.10	1.07 [1.01, 1.13] *P* = 0.01	1.02 [0.96, 1.09] *P* = 0.46	1.06 [1.01, 1.10] *P* = 0.01	1.03 [0.98, 1.08] *P* = 0.23
Hazardous alcohol drinking	0.99 [0.93, 1.06] *P* = 0.83	1.04 [0.96, 1.13] *P* = 0.35	0.98 [0.92, 1.04] *P* = 0.46	1.00 [0.93, 1.07] *P* = 0.98	0.94 [0.90, 0.99] *P* = 0.01	0.93 [0.89, 0.98] *P* = 0.008
Car passenger risk	1.19 [1.11, 1.27] *P* < 0.001	1.15 [1.06, 1.25] *P* = 0.001	1.11 [1.05, 1.18] *P* < 0.001	1.02 [0.95, 1.10] *P* = 0.53	1.08 [1.03, 1.13] *P* = 0.001	1.03 [0.98, 1.09] *P* = 0.26
Cycle-helmet risk	1.02 [0.95, 1.10] *P* = 0.58	1.03 [0.94, 1.12] *P* = 0.58	0.96 [0.90, 1.02] *P* = 0.23	0.91 [0.84, 0.99] *P* = 0.02	1.05 [1.00, 1.10] *P* = 0.06	1.07 [1.01, 1.13] *P* = 0.02
Daily TV viewing (3+ h)	1.30 [1.20, 1.40] *P* < 0.001	1.08 [0.98, 1.17] *P* = 0.11	1.39 [1.29, 1.49] *P* < 0.001	1.27 [1.16, 1.38] *P* < 0.001	1.21 [1.15, 1.27] *P* < 0.001	1.10 [1.04, 1.17] *P* = 0.002
Self-harm	1.10 [1.02, 1.19] *P* = 0.01	1.10 [1.00, 1.21] *P* = 0.06	1.01 [0.95, 1.09] *P* = 0.68	0.94 [0.86, 1.02] *P* = 0.16	1.07 [1.01, 1.12] *P* = 0.02	1.06 [0.99, 1.12] *P* = 0.08
Scooter risks	1.27 [1.17, 1.37] *P* < 0.001	1.17 [1.06, 1.29] *P* = 0.002	1.20 [1.11, 1.29] *P* < 0.001	1.07 [0.97, 1.17] *P* = 0.16	1.14 [1.08, 1.21] *P* < 0.001	1.07 [1.00, 1.14] *P* = 0.04
Sex before age 16 years	1.24 [1.14, 1.34] *P* < 0.001	1.14 [1.03, 1.26] *P* = 0.01	1.22 [1.13, 1.32] *P* < 0.001	1.14 [1.04, 1.25] *P* = 0.005	1.10 [1.04, 1.16] *P* < 0.001	1.02 [0.96, 1.08] *P* = 0.59
Tobacco smoking	1.36 [1.24, 1.50] *P* < 0.001	1.25 [1.11, 1.39] *P* < 0.001	1.25 [1.15, 1.36] *P* < 0.001	1.08 [0.98, 1.19] *P* = 0.14	1.18 [1.11, 1.25] *P* < 0.001	1.08 [1.01, 1.16] *P* = 0.02
Cannabis use	0.96 [0.86, 1.06] *P* = 0.39	1.04 [0.92, 1.17] *P* = 0.57	0.84 [0.77, 0.92] *P* < 0.001	0.80 [0.72, 0.89] *P* < 0.001	1.08 [0.94, 1.08] *P* = 0.78	1.07 [0.99, 1.15] *P* = 0.08
Drug/solvent use	1.04 [0.90, 1.21] *P* = 0.57	1.08 [0.90, 1.30] *P* = 0.40	0.92 [0.81, 1.05] *P* = 0.24	0.85 [0.73, 0.99] *P* = 0.04	1.06 [0.96, 1.16] *P* = 0.26	1.09 [0.97, 1.22] *P* = 0.16
Unprotected sex	1.31 [1.03, 1.68] *P* = 0.03	1.09 [0.81, 1.47] *P* = 0.58	1.54 [1.20, 1.97] *P* < 0.001	1.50 [1.12, 1.99] *P* = 0.006	1.13 [0.96, 1.34] *P* = 0.15	0.99 [0.81, 1.20] *P* = 0.88

Odds ratios with 95% CI indicate linear effect of SES on each behaviour, i.e. the increase in odds of engagement in each risk behaviour for one category change in SES status. For the ‘mutually adjusted’ estimates, the effect of each SES measure has been adjusted for the other two SES measures.

### SES and multiple risk behaviour

The total number of risk behaviours reported was tallied for each participant and grouped for the complete case sample as follows: 0–1 risk behaviour 25.5%, 2–3 behaviours 41.7%, 4–6 behaviours 26.7% and 7+ behaviours 6.1%. Within the larger imputation sample, this distribution changed marginally to reflect the inclusion of participants with larger numbers of risks: 0–1 behaviour 22.0%, 2–3 behaviours 40.6%, 4–6 behaviours 29.0% and 7+ behaviours 8.4%. Table 4 shows the relationship between SES and this composite measure for the imputed data sample. Compared with the highest social class, maternal education or income quintile, the odds of engaging in a greater number of multiple risk behaviours increased for each incremental decrease in social position. For example, with social class, the odds ratio of 1.22 indicates a 22% increase in odds (95% CI: 15–29%), with increases of 15% (95% CI: 9–21%) and 12% (95% CI: 8–16%) for maternal education and income, respectively. Mutual adjustment by the other two measures of SES led to an attenuation of the estimated effects for social class and maternal education; however, the negative association for equivalized income remained strong.

**Table 4:** Relationship between number of risk behaviours and measures of decreasing SES (imputed sample, *n* = 6406)

SES measure	*n*	Number of risk behaviours				Ordinal logistic regression models	
		0–1	2–3	4–6	7–13	Unadjusted OR [95% CI]	Mutually adjusted OR [95% CI]
Parental social class							
Professional	1089	28.8%	42.2%	23.9%	5.1%	1.0 ref	1.0 ref
Managerial	2883	21.9%	40.3%	29.2%	8.7%	1.22 [1.15, 1.29], *P* < 0.001	1.06 [1.02, 1.11], *P* = 0.008
Skilled non-manual	1537	20.8%	40.5%	31.0%	7.8%		
Skilled manual and lower	897	16.4%	39.7%	31.5%	12.5%		
Maternal educational attainment							
Degree	1153	27.4%	40.3%	25.9%	6.4%	1.0 ref	1.0 ref
A-level	1730	23.6%	40.0%	28.6%	7.9%	1.15 [1.09, 1.21], *P* < 0.001	1.04 [0.98, 1.11], *P* = 0.184
O-level	2178	19.8%	41.1%	30.0%	9.1%		
<O-level	1345	19.0%	40.6%	30.7%	9.7%		
Quintiles of household equivalized income							
High	1537	24.9%	41.2%	27.4%	6.6%	1.0 ref	1.0 ref
Middle high	1409	24.8%	39.8%	28.3%	7.1%	1.12 [1.08, 1.16], *P* < 0.001	1.15 [1.07, 1.23], *P* < 0.001
Middle	1281	22.3%	41.1%	27.5%	9.0%		
Middle low	1153	18.6%	40.4%	31.4%	9.7%		
Low	1025	17.4%	40.2%	31.7%	10.7%		

Ordinal regression models under the proportional odds assumption with a linear relationship for each SES predictor variable. *P*-values for contingency tables (not shown) derived from Chi-square statistics were all <0.001. *P*-values shown are derived from Wald tests. Percentages shown are row percents.

For the ‘mutually adjusted’ estimates, the effect of each SES measure has been adjusted for the other two SES measures.

## Discussion

In the ALSPAC cohort at age 15–16 years, the total number of multiple risk behaviours was associated with lower levels of SES. Individual risk behaviours had a more mixed relationship with tobacco smoking, car passenger risk, sex under age 16 years, scooter risk, antisocial and criminal behaviour and TV viewing strongly associated with SES; self-harm, unprotected sex, antisocial and criminal behaviour and cannabis use weakly and inconsistently associated with SES; and little evidence of an association between drug and solvent use, cycling without a helmet and physical inactivity and SES. The findings suggest that interventions designed to reduce engagement in a high number of risky behaviours may need to adopt the approach of proportionate universalism. However, the associations between low SES and high number of risky behaviours are modest. Furthermore, it is known that involvement in one risk behaviour increases the risk of involvement in additional or subsequent risk behaviour. Thus, the associations between individual risk behaviours and SES, or the lack thereof, supports the need for a focus on continued prevention across SES groups.

Analyses of the ALSPAC cohort at the younger age of 13 years and for just two risk behaviours, alcohol and tobacco, found alcohol drinking was more common in young people from higher-income households but less common with higher levels of maternal education,^16^. The current data suggest that this association persists into later adolescence. An inverse socio-economic gradient with tobacco smoking was apparent for all measures of social class. Other studies have examined multiple risk behaviours and SES. A study of tobacco smoking, alcohol drinking, illicit drug use and sexual intercourse among early and late adolescents in Scotland found strong associations between substance use and sexual risk behaviour but that these associations did not differ by social class.^4^ The study did not look at the number of risk behaviours according to social class.

A study of multiple risk behaviours in 14–19-year-olds living in Lao People’s Democratic Republic, where parental education was generally low, found increasing parental education was associated with fewer MRBs.^21^ In contrast, a study of the association of social class with tobacco and cannabis use in 17-year-old adolescents in France, found those from the most affluent families were more likely to be cannabis experimenters and had similar risk of tobacco experimentation, but were less likely to become daily users of both substances during adolescence.^13^ This finding may support the hypothesis that greater access to financial resources with higher SES reduces (or inverts) social patterning. Our data are only partly supportive of this idea, as tobacco, cannabis and other drugs tended to increase with reduced income, while only hazardous alcohol use was more common among the more affluent. A study with 16-year-olds in the Netherlands found no relationship between SES (parental occupation or educational level) and daily smoking, frequent alcohol drinking and ‘soft-drug use’,^10^ but increased participation in sports was associated with higher SES. There was also no relationship between parental SES and complete non-participation in risk behaviours. These findings may support the hypothesis that risk behaviour is part of a general developmental process unrelated to SES. The findings from this study differ from ours; this could be the result of temporal changes, as these Dutch data were collected in the mid-1990s, or it could be because of cultural differences in the social patterning of multiple risk behaviours in the Netherlands compared with a contemporary group in the UK.

The strengths of our study are the assessment of three measures of SES in a contemporary cohort of adolescents, with data available on a wide range of risk behaviours. That the three measures of parental SES were taken during pregnancy and the first few years of life, and therefore may have changed by the time the young people reached age 15/16 years, can be viewed as a limitation, but this study does demonstrate that SES at birth is associated with behaviours at age 15/16 years. A further limitation is that the risk behaviours were all self-reported and reduced to binary variables using cut-off points that were informed by the literature, but different cut-off points could have been selected. It is possible that behaviours showing no relationship with SES in this study would show relationships if examined using a different categorization of engagement in risk behaviour. We also summed the number of behaviours to give a measure of total MRBs, which gives equal weight to each behaviour, yet the impact on adverse outcomes may differ across the behaviours during adolescence and later adulthood.^10^ While the ALSPAC cohort is large, there has been attrition^15^ that, in longitudinal studies, has a tendency to be socially patterned and, therefore, if not accounted for may lead to bias.^22^^,^^23^ ALSPAC participants providing most information tend to be from more educated/affluent families. Our imputed results use a wealth of data collected since recruitment in the prediction models for the missing data to maximize the chances that the assumption that these observations are Missing at Random is satisfied (i.e. that missingness is predictable given the characteristics of the sample).The imputed data suggested that the prevalence of behaviours was higher among participants with missing observations, which was expected given the social patterning of both non-response and many of the outcomes considered, and gives us confidence in the model results. In addition, the association between SES and MRB was found to be robust and was unaltered after imputation. Nevertheless, there remains the possibility that additional, unaccounted for differences between partial and complete ‘non’-responders may affect the generalizeability of these results to the larger ALSPAC sample of >13 000.

There is a need for future epidemiological and qualitative research to investigate the hypotheses posed for different patterns of multiple risk behaviour by SES. The relationship of social status and transition from experimentation to more regular engagement in risk behaviours during adolescence and early adulthood needs to be investigated.

The patterning of behaviours found here suggests that prevention strategies should apply the principal of proportionate universalism with a focus on more deprived populations, within a population-wide strategy, to prevent widening of social inequalities.^14^ However, further understanding is required about the patterning of different multiple risk behaviours and SES. Public health interventions should also be evaluated to assess whether prevention of single risk behaviours or multiple risk behaviours is more effective at reducing harm.

## Supplementary data

Supplementary data are available at *EURPUB* online.

## Funding

The UK Medical Research Council, the Wellcome Trust (WT092731) and the University of Bristol provide core support for ALSPAC. This work was supported by the Centre for the Development and Evaluation of Complex Interventions for Public Health Improvement (DECIPHer), which receives funding from the British Heart Foundation, Cancer Research UK, Economic and Social Research Council (RES-590-28-0005), Medical Research Council, the Welsh Assembly Government and the Wellcome Trust (WT087640MA), under the auspices of the UK Clinical Research Collaboration (R.C., M.H., R.K., M.S.).

*Conflicts of interest*: None declared.

**Key points**

- Risk behaviours in adolescence are common and increase the risk of adult harms.
- Many health behaviours differ by socio-economic status (SES) but social patterning is inconsistent across all risk behaviours. Analyses of multiple risk behaviour rarely examine more than three behaviours.
- We show strong associations between SES and the number of risk behaviours in adolescence. This pattern of SES and risk behaviour suggests that prevention strategies should apply the principal of proportionate universalism with a focus on more deprived populations to prevent widening of social inequalities.
